# Comparative analysis of virulence factors and two-component systems expression in carbapenem-resistant and carbapenem-susceptible *Klebsiella pneumoniae* isolates at a tertiary hospital in Wenzhou, China

**DOI:** 10.1128/spectrum.02030-25

**Published:** 2026-05-26

**Authors:** Hao Chen, Peng Liu, Xiangyang Li, Caixia Liu, Shuo Chen

**Affiliations:** 1 The Second Affiliated Hospital and Yuying Children's Hospital of Wenzhou Medical University 26452, Wenzhou, Zhejiang Province, China; 2 The Second Clinical College of Wenzhou Medical University 26453, Wenzhou, Zhejiang Province, China; 3 Department of Clinical Laboratory, The First Affiliated Hospital of Nanchang University 117970, Nanchang, Jiangxi Province, China; 4 Department of Clinical Laboratory, The Second Affiliated Hospital and Yuying Children’s Hospital of Wenzhou Medical University 26452, Wenzhou, Zhejiang Province, China; 5 Department of Nosocomial Infection Prevention and Control, The First Affiliated Hospital of Wenzhou Medical University 89657, Wenzhou, Zhejiang Province, China; 6 Department of Epidemiology‌, The First Clinical College of Wenzhou Medical University 26453, Wenzhou, Zhejiang, China; Guizhou Medical University, Guiyang, China

**Keywords:** carbapenem-resistant, hypervirulent *Klebsiella pneumoniae*, virulence factor, two-component systems

## Abstract

This study investigated the virulence factors and two-component systems (TCS) expression in carbapenem-resistant *Klebsiella pneumoniae* (CRKP) and carbapenem-susceptible *Klebsiella pneumoniae* (CSKP). A total of 150 CRKP and 87 CSKP clinical isolates were collected from a tertiary hospital. Virulence genes, capsular serotypes, and hypermucoviscosity phenotype were detected. Serum resistance was assessed using serum killing assays, and virulence was preliminarily evaluated using the *Galleria mellonella* larvae lethality assays as a supplement. TCS gene expression (*phoQ*, *rstA*, *rcsB*, and *ompR*) was analyzed by qRT-PCR. The prevalence of hypervirulent *K. pneumoniae* (hvKP, defined by the key virulence gene cluster *rmpA/rmpA2 + iucA + iroB + peg-344*) was significantly lower in CRKP (carbapenem-resistant and hvKP [CR-hvKP], 5.3%) than in CSKP (CS-hvKP, 24.1%; *P* < 0.05). CRKP exhibited lower carriage rates of *rmpA*, *iroB*, *peg-344*, *aerobactin*, *alls*, and *kfu*, but higher rates of *rmpA2* and *ybtS* compared to CSKP. CRKP was more likely to carry incomplete combinations of the key virulence genes than CSKP, with *rmpA/rmpA2 + iucA* being the most common. In CSKP, significant positive correlations were observed among virulence genes, but not in CRKP. In *Galleria mellonella* assays, hvKP and CR-hvKP showed significantly lower survival rates than classical *K. pneumoniae* and non-CR-hvKP (*P* < 0.05). TCS genes (*phoQ*, *rstA*, and *ompR*) were significantly upregulated in hvKP and CR-hvKP (*P* < 0.05). CRKP exhibited reduced virulence gene carriage compared to CSKP, likely due to the loss of virulence genes during plasmid transmission. However, the high prevalence of the key virulence genes in CRKP underscores the potential for CR-hvKP dissemination, necessitating continued surveillance to prevent its spread. The upregulation of *phoQ*, *rstA*, and *ompR* in hvKP is associated with the hypervirulent phenotype, providing correlative clinical evidence that links the expression of these functionally established TCS to hypervirulence in clinical isolates.

**IMPORTANCE:**

Carbapenem-resistant and hypervirulent *Klebsiella pneumoniae* (CR-hvKP) presents a serious clinical threat by combining antibiotic resistance and hypervirulence. This study reveals critical differences in the virulence gene and phenotype between carbapenem-resistant *K. pneumoniae* (CRKP) and carbapenem-susceptible *K. pneumoniae* (CSKP). CRKP has a higher carriage rate of some key virulence genes than CSKP, suggesting region-specific evolutionary adaptations. Although CR-hvKP prevalence was lower than that of CS-hvKP, the persistent detection of key virulence genes in CRKP indicates ongoing dissemination risk, necessitating vigilant surveillance. Furthermore, the concurrent upregulation of two-component system genes (*phoQ*, *rstA*, and *ompR*) in hypervirulent clinical isolates represents a phenotypic signature worthy of further mechanistic exploration.

## INTRODUCTION

*Klebsiella pneumoniae* can be found throughout the human body and the environment and can be categorized into two main types based on pathogenicity: classical *K. pneumoniae* (cKP) and hypervirulent *K. pneumoniae* (hvKP). Unlike cKP, which can colonize healthy individuals without causing symptoms, hvKP poses a significant infection risk even in healthy people, primarily due to its enhanced virulence factors. Studies have demonstrated that up to 50% of young healthy patients infected with hvKP may succumb to the infection (1, 2). Understanding the distinctions between these two forms is crucial for developing targeted treatment strategies and epidemiological surveillance. For instance, a study from South Korea revealed that hvKP accounts for 4.6% of *K. pneumoniae* detected in the intestines of healthy individuals (3). Understanding the virulence factors that contribute to hvKP colonization and infection is an area of ongoing research focus.

In recent years, there has been a significant increase in carbapenem-resistant *K. pneumoniae* (CRKP) and carbapenem-resistant and hypervirulent *K. pneumoniae* (CR-hvKP) (4). Susceptible hvKP can acquire resistance genes through plasmid transfer, while carbapenem-resistant cKP (CR-cKP) can become CR-hvKP by acquiring virulence plasmids from hvKP. This two-way evolutionary mode significantly elevates the risk of large-scale CR-hvKP outbreaks (5, 6). Furthermore, hvKP is more likely to carry *rmpA* or *rmpA2* genes, which regulate capsular polysaccharide (CPS) synthesis (7). Research indicates that hvKP possesses a greater number of iron acquisition systems, including the *kfu* iron uptake system, the *iuc* operon encoding the siderophore aerobactin, the *iro* gene encoding salmochelin, the *ybt* genes encoding yersiniabactin, and the *ent* genes encoding enterobactin (2). Notably, gene sequencing has revealed that genes like *rmpA*, *rmpA2*, *iuc*, *iro*, and *peg-344* can be co-located on the same high virulence plasmid, such as pLVPK (8). Regarding the definition of hvKP, a consensus on the optimal method remains elusive. Currently, numerous studies employ specific combinations of virulence genes, particularly *peg-344*, *iroB*, *iucA*, *rmpA*, and *rmpA2*, as molecular biomarkers for hvKP identification, and this approach is among the most commonly used (9, 10).

It is widely acknowledged that the murine infection model serves as the gold standard for determination. However, this model is relatively cumbersome for large-scale screening. Notably, a recent study by Russo et al., which utilized a mouse pneumonia model and predictive modeling, found that the presence of all five biomarkers (*peg-344*, *iroB*, *iucA*, *rmpA*, and *rmpA2*) was 94% accurate in predicting the hvKP phenotype, and a biomarker count of ≥4 reached 100% sensitivity (11).

The resistance and virulence of *K. pneumoniae* are likely regulated by multiple two-component systems (TCS) (12). Gao et al. demonstrated that the RstA/RstB system in *Escherichia coli* regulates nitrogen metabolism, iron uptake, and acid tolerance systems (13). The PhoP/PhoQ system is the master regulator of lipid A modification and a primary mediator of polymyxin resistance (14). Crucially, McConville et al. demonstrated that such activation of PhoP/PhoQ can synergistically increase bacterial virulence *in vivo* using CRISPR-Cas9 gene editing (15). Its upregulation controls type VI secretion system (T6SS) activity (16). Additionally, Cho et al. suggested a potential link between the PhoP/PhoQ and the synthesis of the *rmpA* factor and capsular polysaccharides (17). Furthermore, the Rcs system has been shown to influence the synthesis of bacterial capsule polysaccharides and is involved in iron acquisition (18). Notably, Wang et al. found that OmpR can regulate mucoviscosity through energy metabolism in *K. pneumoniae* (19).

In this study, the *iucA + iroB + peg-344 + rmpA/rmpA2* key virulence genes cluster was used as the defining marker for hvKP classification. Strains lacking these marker gene clusters were classified as non-hvKP, among which those devoid of any key virulence marker genes were further categorized as cKP. The present study aims to investigate the prevalence of virulence genes among CRKP and carbapenem-susceptible *K. pneumoniae* (CSKP), as well as to compare the expression levels of four TCS genes (*phoQ*, *rstA*, *rcsB*, and *ompR*) in these isolates and assess their correlation with virulence and carbapenem resistance.

## MATERIALS AND METHODS

### Bacterial isolates

A total of 150 CRKP and 87 CSKP isolates were collected and identified from the Second Affiliated Hospital and Yuying Children’s Hospital of Wenzhou Medical University, a teaching hospital in Zhejiang, China, during the period from 2020 to 2021. CRKP isolates were defined as those with a minimum inhibitory concentration (MIC) of ≥4 µg/mL for either imipenem or meropenem, while CSKP isolates were defined as those with the MIC of ≤1 µg/mL for these antimicrobials, according to CLSI guidelines (M100, 2019). Isolates with intermediate resistance (MIC = 2 µg/mL) were excluded from analysis. Based on the detection rate of CRKP in the hospital, the first four isolates of CSKP were stored for use every month (the strains of repeated samples submitted by the same patient were excluded). The carbapenem resistance rate of *K. pneumoniae* fluctuated between 10% and 15% in the institution, prompting the monthly collection of the first four identified CSKP isolates. The species identification and antimicrobial susceptibility testing were performed using MALDI-TOF MS (Bruker Daltonics, Germany) or VITEK 2 (bioMérieux, France).

### Molecular detection of serotype and virulence

The virulence genes (*rmpA*, *rmpA2*, *iucA*, *iroB*, *ybtS*, *kfu*, *peg-344*, *aerobactin*, *iut*, and *alls*) and serotype genes (K1, K2, K5, K20, K54, and K57) of strains were detected by PCR according to the standard conditions described previously (Table S1) (9, 20, 21). DNA sequences were identified using the Nucleotide Basic Local Alignment Search Tool (BLAST) available from the National Center for Biotechnology Information (NCBI).

### Hypermucoviscosity phenotype test

The strains were inoculated onto blood agar plates and incubated at 37°C for 18 h. A single colony was then touched with a sterile bacteriology inoculation loop and gently stretched across the agar surface. A positive string test result, indicative of hypermucoviscous *K. pneumoniae* (hmKP), was defined by the formation of a viscous string exceeding 5 mm in length upon lifting the loop. Strains that did not meet this criterion were classified as non-hmKP.

### Serum killing assay

Isolates for serum killing assays were selected with consideration of specimen type distribution. Among CRKP, sputum accounted for 73.3%, blood for 10.0%, and other sources 16.7%. Among CSKP, sputum accounted for 81.6%, blood for 4.6%, and other sources for 13.8%. Accordingly, we selected five CS-hvKP, five CS-cKP, and five CR-cKP, based on the distribution of the above specimens. For the CR-hvKP category, all eight available strains were included (three from blood and five from sputum). Serum from healthy physical examinees was stored at −80°C. A 0.5 McFarland bacterial solution, approximately 1.5 × 10^8^ CFU/mL, was prepared using PBS and diluted to a final concentration of 1 × 10^6^ CFU/mL. A total of 50 µL of bacterial suspension and 150 µL of healthy human serum were dispensed into microtitration trays, mixed, and then incubated at 37°C. To assess viability, the mixture was streaked onto blood agar plates immediately (time zero), after 1 h, after 2 h, and finally after 3 h. Each isolate was tested three times (22).

### *Galleria mellonella* larvae lethality assay

The strains subjected to serum resistance testing were also evaluated for pathogenicity using the *Galleria mellonella* larvae lethality assay, ensuring consistency across experiments. Virulence assays were conducted using the larval lethality of *Galleria mellonella* as an infection model, with slight modifications from a previously described method (23). Larvae weighing approximately 270–330 mg were selected and maintained at 20°C in darkness with unrestricted access to food. Overnight cultures of *K. pneumoniae* strains on blood agar plates were diluted in PBS to a final concentration of 1 × 10^6^ CFU/mL. A 5 μL aliquot of the bacterial suspension was plated for colony counting to ensure consistency in bacterial inoculum. After surface disinfection with 70% ethanol, larvae were gently injected with 10 μL of bacterial suspension into the last right proleg. Seven *Galleria mellonella* larvae were injected with each test isolate. Following injection, larvae were placed on plates with clean, moist gauze in a dark environment at 37°C. *K. pneumoniae* ATCC70063 and *K. pneumoniae* NTUH-K2044 were used as controls. Larval activity was monitored and recorded at 24 h, 48 h, 72 h, and 96 h post-injection.

### RNA isolation and qRT-PCR

Total RNA from the *K. pneumoniae* isolates used in the phenotypic assays was extracted using Invitrogen TRIzol reagent (Thermo, USA). The reverse transcription reaction was performed in 20 μL mixtures using PrimeScript RT Reagent Kit Perfect Real Time (Takara, Japan). The expression levels of two-component systems, including *phoQ*, *rstA*, *rcsB*, and *ompR*, were determined by qRT-PCR (Table S2). The transcription level was analyzed by the cycle threshold method (2^−△△CT^), normalized to the expression level of cKP-1, with *rpsL* as the internal reference gene. cKP-1 was selected as the calibrator strain because: (i) it lacked all 10 key virulence genes tested (Table S1), confirming its classification as a classical strain; (ii) it showed stable ΔCt values across technical and experimental replicates; (iii) it exhibited baseline virulence in the *Galleria mellonella* infection model, consistent with its classical phenotype. The Mann-Whitney *U*-test was used for statistical comparison between groups.

## RESULTS

### Prevalence of virulence genes among CRKP and CSKP

**Fig 1:**
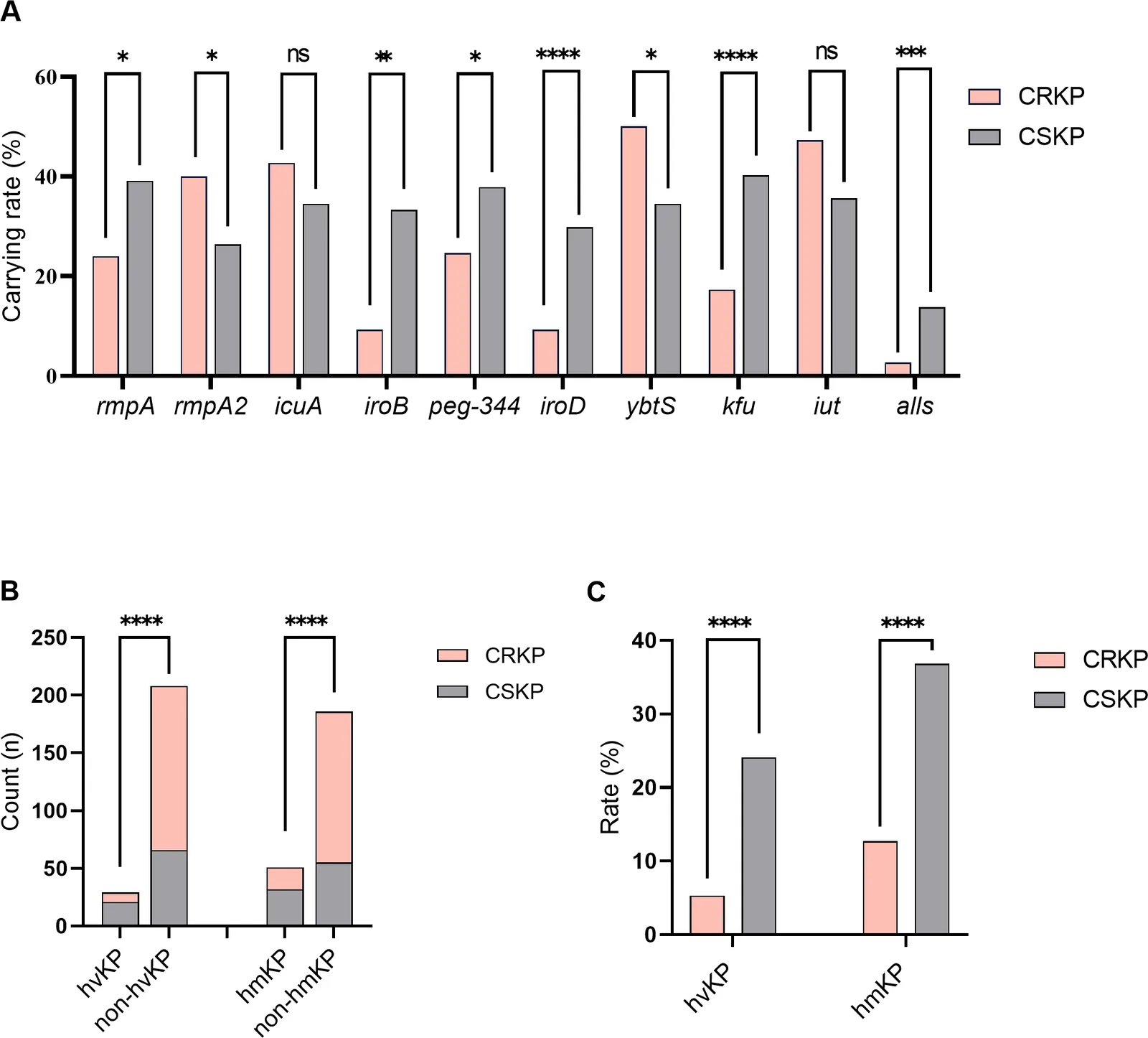
Differential distribution of virulence factors in 150 CRKP strains and 87 CSKP strains. (**A**) The carrying rate detection between CRKP and CSKP, *P < 0.05, **P < 0.01, ***P < 0.001, and ****P < 0.0001; ns, not significant. (**B**) Count of CRKP and CSKP in hvKP and hmKP, ****P < 0.0001. (**C**) Proportion of CRKP and CSKP within hvKP and hmKP, ****P < 0.0001.

The positive rates of the 10 virulence genes (*rmpA*, *rmpA2*, *iucA*, *iroB*, *peg-344*, *aerobactin*, *ybtS*, *kfu*, *iut*, and *alls*) in 237 *K. pneumoniae* strains were 29.5%, 35.0%, 39.7%, 18.1%, 29.5%, 16.9%, 44.3%, 25.7%, 43.0%, and 6.8%, respectively. The distribution of virulence genes was detailed in Table 1. Notably, 2.67% (4/150) of CRKP isolates and 23.0% (20/87) of CSKP isolates lacked any of these virulence genes (Fisher’s exact test, *P* < 0.0001). CRKP exhibited significantly lower positive rates of the genes *rmpA*, *iroB*, *peg-344*, *aerobactin*, *alls*, *and kfu* compared to CSKP (*P* < 0.05), with rates of 24.0%, 9.3%, 24.7%, 9.3%, 2.7%, and 17.3%, respectively. Additionally, the detection rates of *rmpA2* and *ybtS* in CRKP were 40.0% and 50.0%, significantly higher than that in CSKP (*P* < 0.05; Fig. 1A).

**TABLE 1:** Differences in virulence gene carrying rate in CRKP and CSKP

Gene	CRKP*, n* (%)	CSKP*, n* (%)	*χ*^2^ value	*P*
*rmpA*	36 (24.0)	34 (39.1)	6.017	0.014
*rmpA2*	60 (40.0)	23 (26.4)	4.451	0.035
*iucA*	64 (42.7)	30 (34.5)	1.541	0.241
*iroB*	14 (9.3)	29 (33.3)	21.356	<0.0001
*peg-344*	37 (24.7)	33 (37.9)	4.655	0.031
*aerobactin*	14 (9.3)	26 (29.9)	16.578	<0.0001
*ybtS*	75 (50.0)	30 (34.5)	5.373	0.02
*kfu*	26 (17.3)	35 (40.2)	15.103	<0.0001
*iut*	71 (47.3)	31 (35.6)	3.075	0.079
*alls*	4 (2.7)	12 (13.8)	10.82	0.0009

Among the 150 CRKP examined, only 5.3% (8/150) were classified as CR-hvKP. In contrast, 24.1% (21/87) of CSKP were identified as CS-hvKP. The probability of CSKP being hvKP was significantly higher than that of CRKP (*χ*² =18.11, *P* < 0.001; Fig. 1B and C). At the same time, we found that the probability of carrying incomplete combinations of key virulence marker genes was higher in CRKP than in CSKP. Specifically, 50.7% (76/150) of CRKP carried one to three of these critical virulence marker genes, whereas only 26.4% (23/87) of CSKP did. In CRKP, the most prevalent combination of the aforementioned key virulence genes was *rmpA/rmpA2 + iucA*, accounting for 20.0% (30/150). The specific distribution of the key virulence genes can be found in Table 2.

**TABLE 2:** The specific distribution of the key virulence genes in CRKP and CSKP

Combination of different key virulence marker genes	CRKP*, n* (%)	CSKP*, n* (%)
*iroB*	1 (0.7)	1 (1.1)
*iucA*	4 (2.7)	2 (2.3)
*peg-344*	4 (2.7)	4 (4.6)
*iroB + peg-344*	1 (0.7)	1 (1.1)
*iroB + iucA + peg-344*	1 (0.7)	0 (0.0)
*rmpA/rmpA2*	10 (6.7)	3 (3.4)
*rmpA/rmpA2 + iroB*	0 (0.0)	1 (1.1)
*rmpA/rmpA2 + iucA*	30 (20.0)	2 (2.3)
*rmpA/rmpA2 + peg-344*	2 (1.3)	1 (1.1)
*rmpA/rmpA2 + iroB + peg-344*	3 (2.0)	3 (3.4)
*rmpA/rmpA2 + iroB + iucA*	1 (0.7)	2 (2.3)
*rmpA/rmpA2 + iucA + peg-344*	19 (12.7)	3 (3.4)
*rmpA/rmpA2 + iroB + iucA + peg-344* (hvKP)	8 (5.3)	21 (24.1)
Carry none (cKP)	66 (44.0)	43 (49.4)

### Detection of hypermucoviscosity phenotype

A total of 21.5% (51/237) of *K. pneumoniae* strains tested positive in the string test and were classified as hmKP. The prevalence of hmKP was significantly lower in CRKP (19/150, 12.7%) compared to CSKP (32/87, 36.8%; *χ*² =18.96, *P* < 0.001; Fig. 1B and C). Among the 51 hmKP, 39.2% (20/51) were identified as hvKP, characterized by the presence of key virulence marker genes. However, no statistically significant difference was observed in the association between hmKP and hvKP within CRKP and CSKP. Furthermore, no significant correlation was found between the hmKP phenotype and the presence of the *rmpA*/*rmpA2* genes.

### The virulence of different capsule serotype isolates

The capsular serotype of the 150 CRKP revealed the following distribution: K1 (10, 6.6%), K2 (2, 1.3%), K20 (4, 2.7%), and K5 (1, 0.7%). No strains with K54 or K57 capsule serotypes were detected. Among CSKP, the distribution was as follows: K1 (9, 10.3%), K2 (4, 4.6%), K54 (3, 3.4%), K5 (1, 1.1%), and K20 (1, 1.1%). Statistical analysis indicated no significant difference in capsular serotype distribution between CRKP and CSKP (Table S3). The distribution of hvKP and non-hvKP across capsular serotypes (K1, K2, K5, K20, K54, and others) showed significant differences (*χ*² = 58.06, *P* < 0.001). Specifically, the prevalence of hvKP was significantly higher in the K2 (4, 66.7%) and K1 (10, 52.6%) serotypes compared to K20 (2, 40.0%), K54 (5, 33.3%), and non-typeable or other serotypes (12, 5.9%). Further analysis revealed that the proportion of the K1 serotype was significantly higher in hvKP (10/29, 34.48%) than in non-hvKP (9/208, 4.3%). Similarly, the proportion of the K2 serotype was significantly higher in hvKP (4/29, 13.8%) compared to non-hvKP (2/208, 1.0%). Both K1 and K2 serotypes were significantly overrepresented in hvKP compared to non-hvKP (K1: Fisher’s exact test, *P* < 0.0001; K2: Fisher’s exact test, *P* = 0.0012).

**Fig 2:**
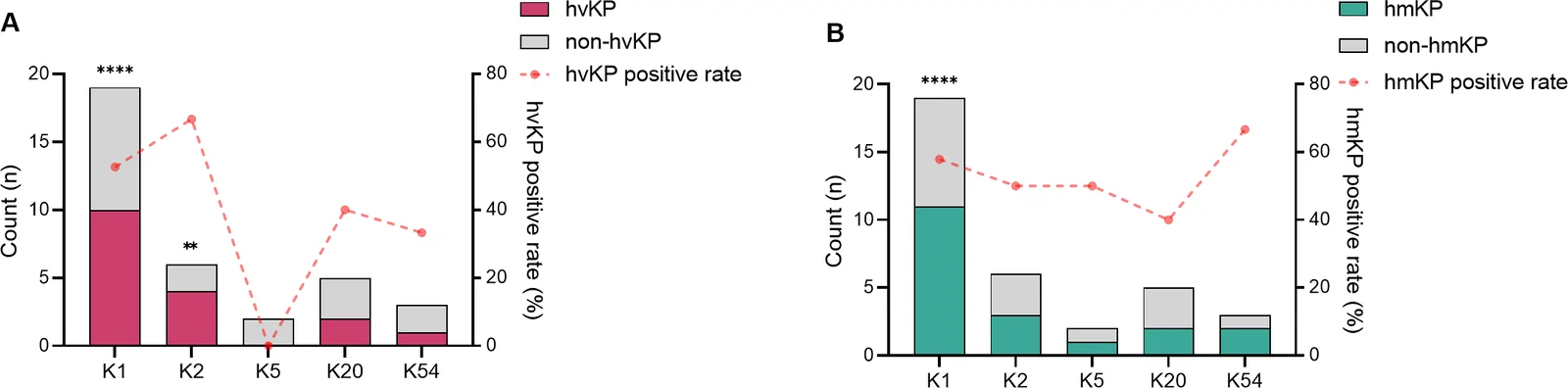
Detection rates of hmKP and hvKP in capsule serotypes. (**A**) The detection rate of hvKP, **P < 0.01 and ****P < 0.0001. (**B**) The detection rate of hmKP, ****P < 0.0001.

The prevalence of hmKP, similar to that of hvKP, showed significant differences in distribution across capsular serotypes (*χ*² = 27.22, *P* < 0.001). Among hmKP, K54 exhibited the highest prevalence of hmKP (2, 66.7%), followed by K1 (11, 57.9%), K2 (3, 50.0%), K5 (1, 50.0%), and non-typeable or other serotypes (32, 15.8%). Further analysis revealed that the proportion of hmKP in the K1 serotype (11/51, 21.6%) was significantly higher than that in non-hmKP (8/186, 4.3%; Fisher’s exact test, *P* < 0.001). The positivity rates of hvKP and hmKP across different capsular serotypes are presented in Fig. 2.

### Virulence gene distribution correlation

**Fig 3:**
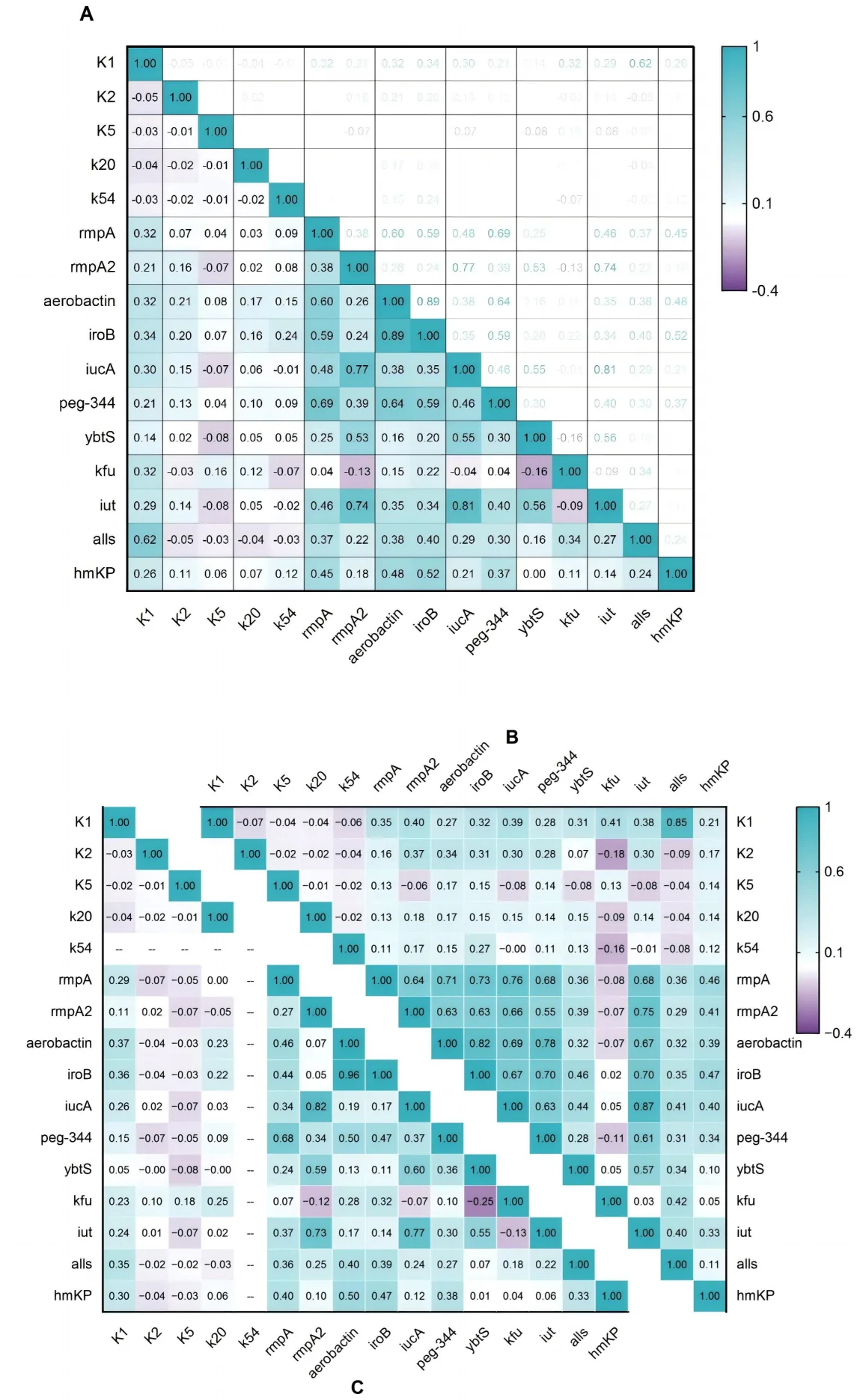
Gene correlation analysis. (**A**) Correlation analysis of virulence genes in strain 237 KP strains. (**B**) Correlation analysis of virulence genes in 150 CRKP strains. (**C**) Correlation analysis of virulence genes in 87 CSKP strains. A correlation coefficient greater than 0.7 is considered indicative of a strong correlation.

According to the heatmap of virulence gene distribution correlation (Fig. 3A), *iroB* exhibited a very strong positive correlation with *aerobactin*. In addition, *iut* showed strong positive correlations with both *rmpA2* and *iucA*, while *rmpA* and *peg-344* were moderately positively correlated. No other notable negative correlations were observed among these virulence genes.

In CSKP, the genes *rmpA*, *rmpA2*, *iucA*, *iroB*, *peg-344*, and *aerobactin* tended to coexist, showing significant correlations (Fig. 3B). Additionally, the *iut* gene also exhibited strong positive correlations with most of these genes (*rmpA*, *rmpA2*, *iucA*, *iroB*, and *aerobactin*), but its correlation with *peg-344* was moderate. Furthermore, the K1 capsular serotype gene showed a very strong positive correlation with *alls*. In contrast, in CRKP, only *rmpA2*, *iucA*, and *iut* were highly positively correlated (Fig. 3C). Notably, no significant correlation was observed between hmKP and the aforementioned virulence genes in either CRKP or CSKP.

### Serum killing assay in different *K. pneumoniae* strains

**Fig 4:**
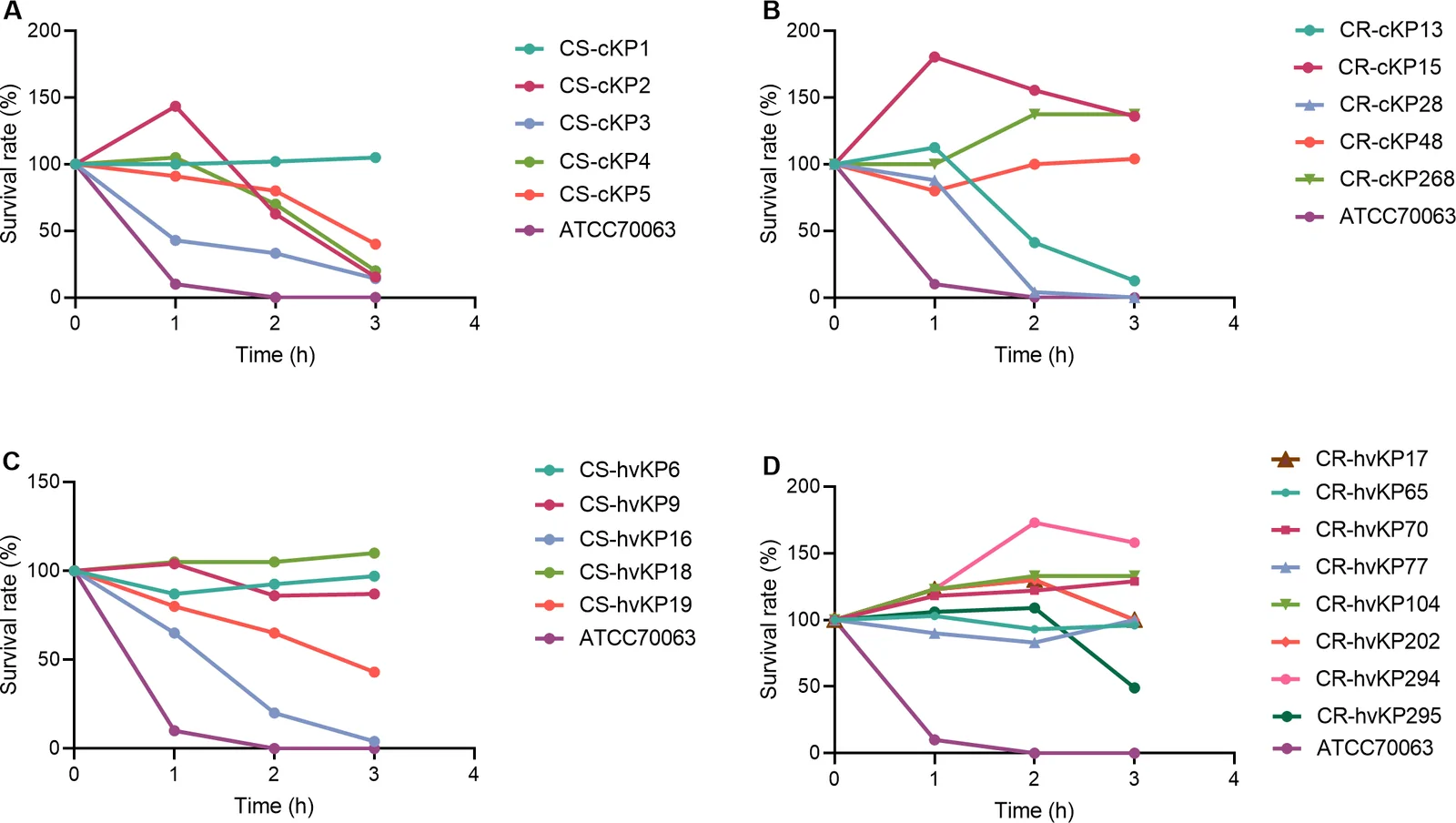
Growth of *K. pneumoniae* grown in human serum at 0 h, 1 h, 2 h, and 3 h. (**A**) Growth of CS-cKP. (**B**) Growth of CR-cKP. (**C**) Growth of CS-hvKP. (**D**) Growth of CR-hvKP. *K. pneumoniae* ATCC70063 as a control strain.

Serum resistance was compared among the selected strain sets (five CS-hvKP, five CS-cKP, five CR-cKP, and eight CR-hvKP) as described in Materials and Methods (Fig. 4). Statistical analysis revealed no significant differences in serum resistance distribution between the following groups: hvKP and cKP (10/13 vs. 4/10 resistant; *χ*² = 2.68, *P* = 0.102), CRKP and CSKP (10/13 vs. 4/10 resistant; *χ*² = 2.68, *P* = 0.102), and CR-hvKP and non-CR-hvKP (7/8 vs. 7/15 resistant; *χ*² = 2.94, *P* = 0.086).

### Pathogenicity in *Galleria mellonella* model

**Fig 5:**
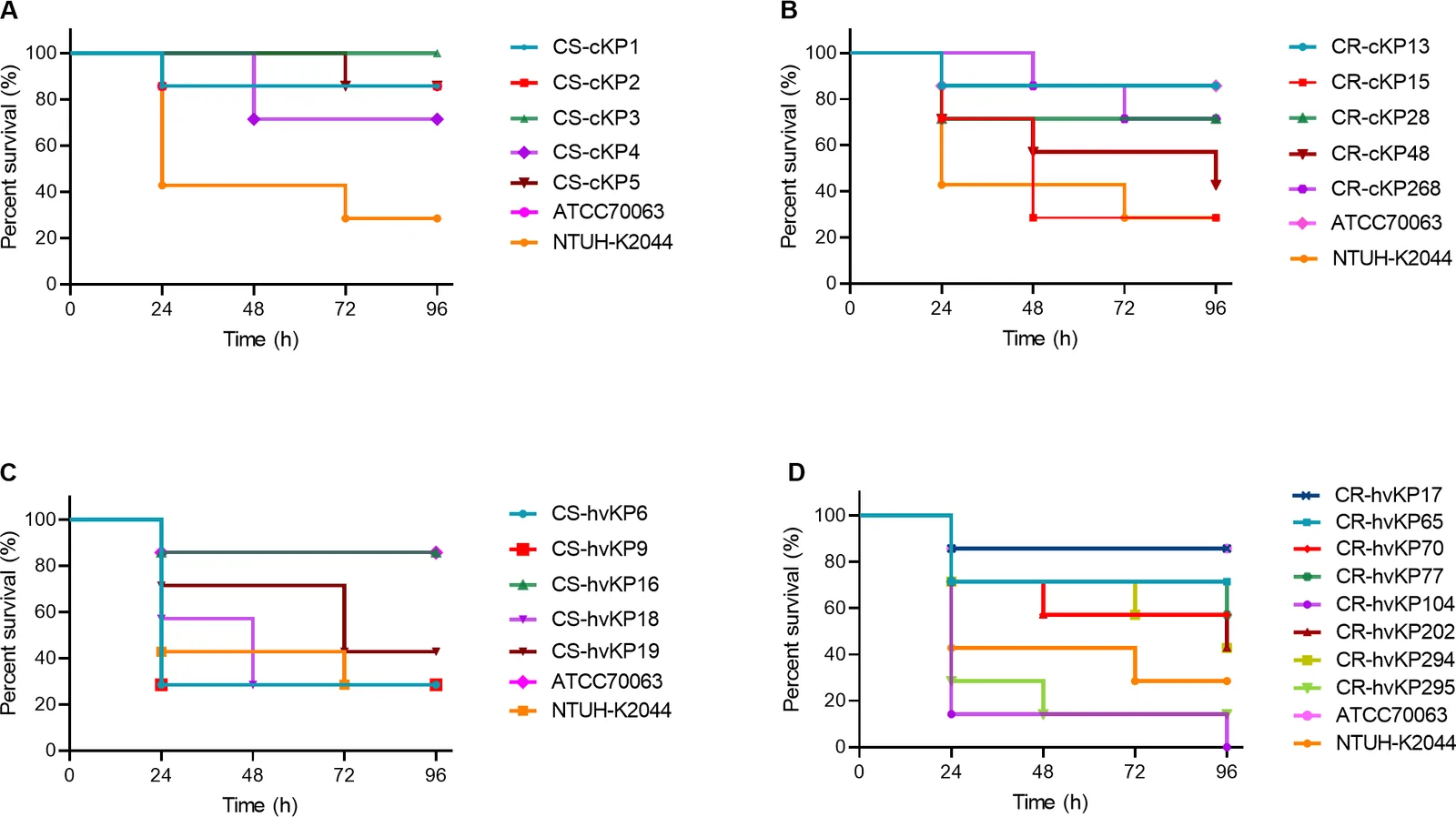
Survival curves of *Galleria mellonella* larvae lethality assay. (**A**) Survival curves of *Galleria mellonella* larvae with CS-cKP. (**B**) Survival curves of *Galleria mellonella* larvae with CR-cKP. (**C**) Survival curves of *Galleria mellonella* larvae with CS-hvKP. (**D**) Survival curves of *Galleria mellonella* larvae with CR-hvKP. *K. pneumoniae* ATCC70063 and *K. pneumoniae* NTUH-K2044 as control strains.

Survival curves of the *Galleria mellonella* larvae lethality assay are shown in Fig. 5. A total of 7 larvae were injected per test isolate. At 96 h post-infection, the survival rate of larvae inoculated with the 11 hvKP and the 7 cKP was significantly lower than that of larvae inoculated with the control strain ATCC 700603. Notably, the survival rate of larvae inoculated with the 2 CR-hvKP was even lower than that of the reference strain NTUH-K2044, with the lowest observed survival rate reaching 0.0%. Larvae infected with hvKP showed significantly lower survival than those infected with cKP (41/91 [45.1%, 95% CI: 34.8%–55.6%] vs. 51/70 [72.9%, 95% CI: 61.8%–82.1%]; *χ*² = 12.49, *P* < 0.001). Similarly, larvae infected with CR-hvKP exhibited lower survival compared to those infected with non-CR-hvKP (26/56 [46.4%, 95% CI: 33.0%–60.3%] vs. 66/105 [62.9%, 95% CI: 53.0%–72.0%]; *χ*² = 4.03, *P* = 0.045). However, no significant difference was observed between CRKP and CSKP (47/91 [51.6%, 95% CI: 41.0%–62.2%] vs. 45/70 [64.3%, 95% CI: 52.0%–75.5%]; *χ*² = 2.58, *P* = 0.108).

### Relative expression of two-component systems

**Fig 6:**
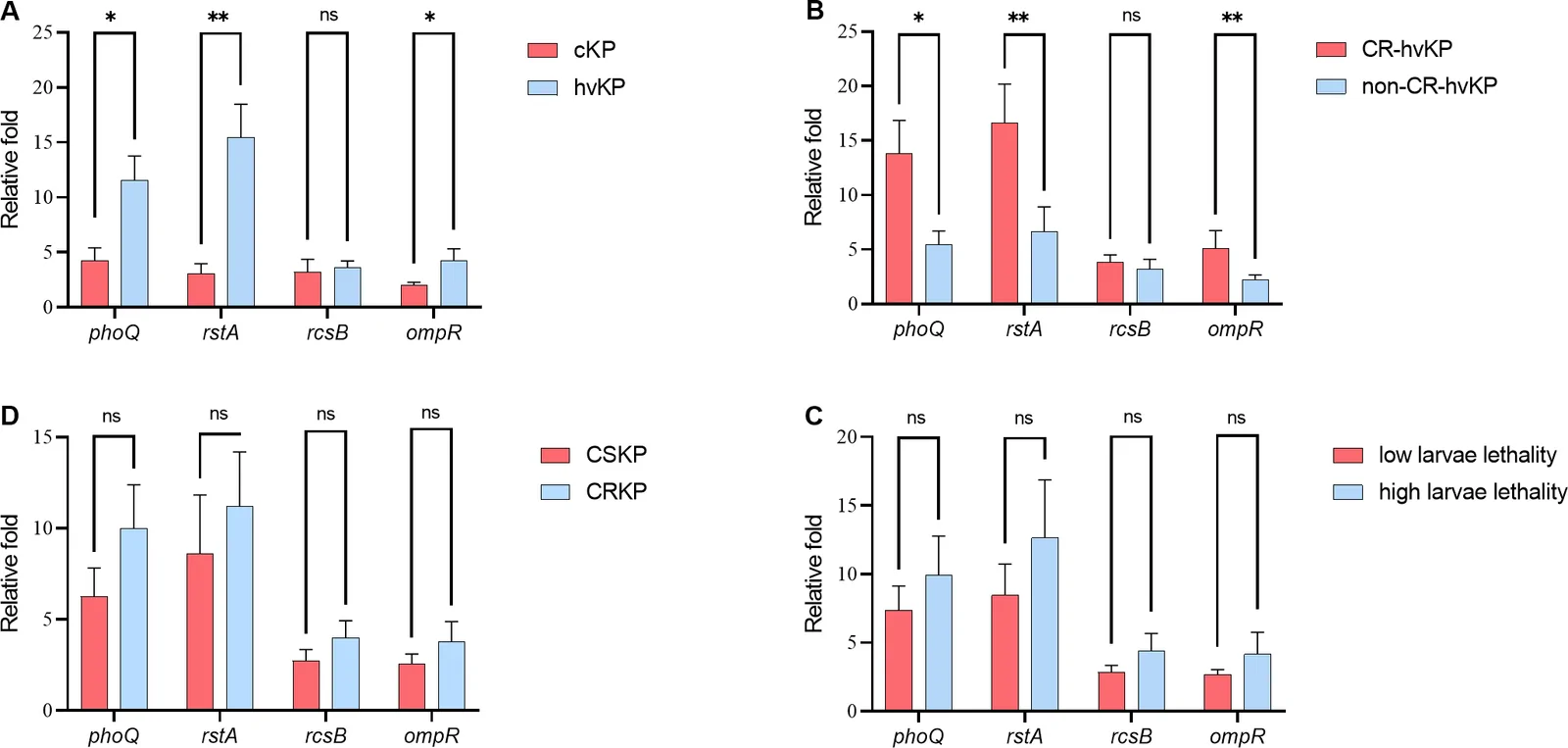
Relative expression of *phoQ*, *rstA*, *rcsB*, and *ompR*. (**A**) The relative expression between cKP and hvKP, *P < 0.05 and **P < 0.01. (**B**) The relative expression between CR-hvKP and non-CR-hvKP, *P < 0.05 and **P < 0.01. (**C**) The relative expression between low larvae lethality and high larvae lethality of *Galleria mellonella* larvae. (**D**) The relative expression between CRKP and CSKP. ns, not significant.

The relative expression levels of the two-component systems in the tested isolates are presented in Fig. 6. The median transcript level of *phoQ* was 9.907-fold higher in the hvKP group compared to 4.109-fold in the cKP group (*z* = −2.419, *P* = 0.016). Similarly, *rstA* transcript levels were significantly higher in hvKP (median = 13.105-fold) than in cKP (median = 2.571-fold; *z* = −3.093, *P* = 0.002), and *ompR* transcript levels were significantly higher in hvKP (median = 3.735-fold) compared to cKP (median = 1.950-fold; *z* = −2.233, *P* = 0.026). However, no significant difference was observed in *rcsB* expression. Significant differences in expression levels were observed for *phoQ*, *rstA*, and *ompR* between hvKP and cKP, as well as between CR-hvKP and non-CR-hvKP (Fig. 6A and B). In contrast, no significant differences in expression were found between the isolates with *Galleria mellonella* survival rates below and above 50% after 96 h of infection, as well as between CRKP and CSKP (Fig. 6C and D).

## DISCUSSION

The prevalence of virulence genes in *K. pneumoniae* exhibits marked geographical heterogeneity, as evidenced by a nationwide survey reporting hvKP rates ranging from 73.9% in Wuhan to 8.3% in Zhejiang Province (24). This regional variation is also pronounced among CRKP (4, 25). Our findings align with this pattern: the CR-hvKP rate in Wenzhou (5.3%) was higher than that in Ningbo (0.7%), a city located only 200 km away, yet significantly lower than the rate observed in Nanning (39.1%) (10, 26). This disparity in CR-hvKP prevalence correlates directly with differences in the carriage rates of key virulence genes among regional CRKP populations. For instance, the *rmpA* carriage rate in our CRKP (24.0%) was lower than that reported in Nanning (40.6%), while the *iroB* rate (9.3%) was notably higher than in Ningbo (1.3%). These observations suggest that the geographical variation in CR-hvKP prevalence fundamentally reflects differences in the inherent virulence gene repertoires of the predominant CRKP circulating in different hospital ecosystems.

A distinctive finding of our study was the significantly higher carriage of *rmpA2* (40.0%) and *ybtS* (50.0%) in CRKP compared to CSKP (Table 1). This pattern is not isolated, and a study from Ningbo also reported a higher *ybtS* carriage rate in CRKP (75.0%) than in CSKP (47.9%) (26). This further supports a potential common trend where CRKP may selectively retain or acquire this siderophore system. Notably, this high carriage rate stands in contrast to a genomic survey of *K. pneumoniae* species complex isolates collected from the same hospital in a different period, which reported significantly lower overall rates for *rmpA2* and *ybtS* (27). The concurrent elevation of these two genes in our CRKP may be mechanistically linked. Notably, *rmpA2* is a key regulator of CPS, enhancing immune evasion and colonization (28). Exposure to antibiotic stress can co-upregulate the expression of both *rmpA2* and carbapenemase genes in CRKP, suggesting a coordinated adaptive response (29). The *ybtS* gene, part of the yersiniabactin siderophore system, is crucial for iron acquisition under host-limiting conditions. Given that over half of hospitals with carbapenemase-positive isolates likely experience nosocomial clonal spread (30), we hypothesize that the co-enrichment of *rmpA2* and *ybtS* observed here may signal the successful expansion and transmission of one or more CRKP clones possessing this combination of traits, which likely confers a fitness advantage in the local hospital environment. Supporting this, high virulence gene carriage and clonal spread in intensive care units have been concurrently documented in CRKP (31). Whether driven primarily by the intrinsic fitness of the bacterial clone or facilitated by transient lapses in infection control, the localized emergence and persistence of such strains highlight their adapted potential for transmission within a specific healthcare setting. This localized clonal dynamic provides a micro-evolutionary basis for the macro-scale regional differences in CR-hvKP prevalence.

The proportions of both hvKP and cKP in CRKP were significantly lower than those in CSKP. Furthermore, the higher prevalence of non-hvKP CRKP carrying incomplete combinations of key virulence marker genes compared to CSKP suggests potential gene loss during plasmid transfer or the modular acquisition of virulence and resistance determinants. Although the current prevalence of CR-hvKP remains low, the fact that 20% of CRKP carried the *rmpA*/*rmpA2 + iucA* combination underscores the importance of vigilance. Moreover, the persistent detection of CRKP carrying key virulence genes indicates an ongoing risk of dissemination, necessitating close attention and proactive surveillance.

In our study, 12.7% of CRKP exhibited a positive string test, which is consistent with the prevalence of hvKP among CRKP reported in previous studies. Notably, four of these string test-positive strains lacked the hypervirulent biomarker genes, further supporting the notion that the string test is not a reliable method for hvKP identification. Although earlier studies suggested a correlation between hmKP and the presence of *rmpA*/*rmpA2* genes, our study did not find a significant association between these factors.

The debate regarding the virulence of K1/K2 strains continues. While some studies suggest that non-K1/K2 strains can also exhibit high virulence, potentially independent of K1/K2 typing (32), our study observed a significantly higher prevalence of hvKP among K1 and K2 serotypes compared to other serotypes. Additionally, K1 and K2 serotypes were more prevalent in hvKP than in non-hvKP. We also found a stronger association between the K1 serotype and the carriage of key virulence genes, which may contribute to its enhanced virulence. However, although differences in hvKP and hmKP prevalence were observed between CRKP and CSKP, no significant differences in capsular serotype distribution were detected between CRKP and CSKP in our study. This contrasts with findings from a study in Ningbo, which reported that the detection rate of CSKP was higher than that of CRKP for all capsule serotypes (33). In the correlation analysis, no significant association was observed between K1/K2 serotypes and key virulence marker genes, except for a positive correlation between K1 and *alls*, consistent with the findings of Min Jiang et al. (26). In CSKP, the genes *rmpA*, *rmpA2*, *iucA*, *iroB*, *peg-344*, and *aerobactin* tended to coexist, showing significant positive correlations, which is consistent with the findings of Min Jiang et al. However, it is noteworthy that the strength of these correlations appears somewhat attenuated in our cohort. This observation further supports the notion of regional variation in virulence gene association patterns, possibly reflecting differences in the prevalent plasmid variants or host bacterial genetic backgrounds that influence the stability and linkage of these virulence determinants. In contrast, this correlation pattern was largely absent in CRKP, likely due to the loss of these gene clusters during the acquisition of carbapenem resistance determinants, or vice versa. Notably, *aerobactin*, which is also referred to as *iroD* in some literature, was highly correlated with *iroB*.

Interestingly, our study revealed no significant difference in serum resistance between hvKP and cKP or between CRKP and CSKP, though the limited subgroup sample sizes should be considered when interpreting these negative findings. While serum contains complement proteins and lysozyme with bactericidal activity, it lacks immune cells like phagocytes that are crucial for bacterial clearance.

The *Galleria mellonella* infection experiments provided supportive evidence that hvKP, regardless of carbapenem resistance, possessed higher pathogenic potential. Compared to cKP and non-CR-hvKP, hvKP and CR-hvKP showed significantly lower larval survival rates. However, it is important to acknowledge the limitations of this model. Thomas et al. systematically evaluated the *G. mellonella* model using well-characterized hvKP and cKP and found substantial overlap in individual strain virulence, with some cKP exhibiting higher lethality than hvKP (34). This aligns with our observation that one cKP in our study, isolated from a bloodstream infection, also exhibited high lethality, reinforcing that the *G. mellonella* model may not reliably distinguish between hvKP and cKP at the individual strain level. Despite these limitations, other studies have supported the utility of the *G. mellonella* model for assessing relative virulence potential, particularly when comparing strains with clear phenotypic differences (35). These findings suggest that while the *G. mellonella* model can provide supportive evidence, it should not be used as a definitive tool for validating the hypervirulence phenotype of individual isolates. The murine infection model remains the acknowledged gold standard for defining hypervirulence. A pivotal recent study systematically evaluated biomarkers against a mouse pneumonia model and found that the presence of all five key biomarkers predicted the hvKP phenotype with 94% accuracy (11). This provides strong validation for the molecular definition employed in our study. It is worth noting that results may vary with different infection routes (intraperitoneal vs. pulmonary), underscoring the complexity of virulence assessment across models. Additionally, Tian et al. suggested that hvKP carrying KPC plasmids could hardly exhibit both hypervirulence and carbapenem resistance simultaneously, as this could lead to a significant reduction in the original hypermucoviscosity phenotype while maintaining serum resistance and high toxicity (36).

TCS are ubiquitous in bacteria and crucial for environmental sensing and adaptive responses. While most prior functional studies of TCS in reference or engineered strains have employed genetic knockout models, we investigated their expression profiles in a diverse collection of clinical isolates. We observed significantly higher expression of *phoQ*, *rstA*, and *ompR* in hvKP compared to cKP, with robust statistical support (*P* < 0.05, statistical power >80% for *phoQ* and *ompR*). These findings align with and extend previous mechanistic studies. The PhoP/PhoQ system has been linked to capsule synthesis and T6SS activity (15, 16). This connection gains further context from the recent observation of Chen et al. (29). Integrating these findings, it is plausible to hypothesize that PhoQ may serve as a regulatory nexus, responding to antibiotic stress or host-derived signals to coordinately influence both virulence (via RmpA/RmpA2) and resistance. While our data show an association between *phoQ* expression and the hvKP genetic profile, this proposed regulatory interplay warrants direct experimental validation in future studies.

OmpR has been demonstrated to promote lung infection through the transcriptional regulation of key virulence factors (37). Specifically, Wang et al. demonstrated that OmpR can enhance virulence, such as biofilm formation and serum resistance, by repressing Acetylated CPS Esterase in a mouse pneumonia model (38). Supporting this, a subsequent study using an *ompR* knockout confirmed that its absence significantly attenuates mortality in a mouse lung infection model (19). Regarding RstA, although less studied in *K. pneumoniae*, knockout and complementation experiments have confirmed its functional role. Zhang et al. constructed *rstA* knockout and found that the mutant exhibited reduced serum resistance *in vitro* and attenuated virulence in both *Galleria mellonella* and mouse models (39). Our findings demonstrate a significant association between *rstA* and hvKP virulence (*P* = 0.002), with the high statistical power (96%) of this analysis further supporting the reliability of this observation.

Therefore, our observation of their upregulation in hvKP provides clinical correlative evidence that aligns with these established functional roles, positioning PhoQ, RstA, and OmpR as high-priority candidates in hvKP isolates. It is important to note that the observed upregulation represents a correlative association, not direct mechanistic proof of causality. While these findings strongly suggest a potential regulatory link between these TCS and the hypervirulent phenotype, definitive evidence requires functional genetic validation through gene knockout and complementation studies. The elevated expression of these TCS genes could be either a cause or a consequence of the hypervirulent state, or both might be regulated by a common factor, analogous to the stress-induced upregulation of *rmpA2* (29). Our correlative data cannot distinguish between these possibilities, which should be addressed in future mechanistic studies.

For RcsAB, Su et al. reported the involvement of RcsAB in regulating *K. pneumoniae* virulence through the modulation of multiple genes, including *rmpA* (40). Similarly, Peng et al. demonstrated the impact of RcsAB on virulence, biofilm formation, and CPS production using gene knockout approaches (41). In contrast, our study did not detect significant differences in *rcsB* gene expression levels between hvKP and cKP overall. However, this discrepancy may reflect methodological differences (knockout vs. clinical isolates) or the limited statistical power of *rcsB* gene expression levels. This negative result should be interpreted cautiously. The knockout models’ clear phenotypic effects suggest that RcsB’s regulatory role might manifest primarily under specific conditions or through post-transcriptional mechanisms not captured by expression analysis alone.

Recent work by Song et al. demonstrated that canonical virulence factors, including the enterobactin and yersiniabactin siderophore clusters, were significantly upregulated in high-burden murine infection models compared to low-burden conditions (42). Our re-analysis of their data set (GSE289556) revealed a similar pattern for key TCS regulators: expression levels of *phoQ*, *rcsB*, *rstA*, and *ompR* were consistently higher under high-burden infection conditions *in vivo* (Fig. S1). Moreover, the expression of these TCS genes increased progressively over time, suggesting a dynamic bacterial response to sustained infection pressure (Tables S4 and S5). It is important to note that this data set compares infectious burden within a single multidrug-resistant strain, rather than directly comparing hvKP and cKP pathotypes. As such, it provides indirect rather than direct evidence for TCS involvement in the hypervirulent phenotype. The observed upregulation under high infectious burden nonetheless offers a supportive context for our clinical findings. Such direct comparative transcriptomic data sets between hvKP and cKP clinical isolates under controlled *in vitro* conditions remain scarce in public repositories.

Furthermore, in our cohort, the expression levels of individual TCS genes showed no significant correlation with high mortality rates in the *Galleria mellonella* model. This is intriguing, given that knockout studies of these same TCS have demonstrated their clear impact on virulence in this model (17, 19, 39). This apparent discrepancy reinforces the notion that in clinical hvKP isolates, virulence is likely governed by the coordinated action of multiple regulatory systems, rather than the expression level of any single TCS. The functional presence of these TCS may be essential (as shown by knockouts), but their quantitative expression variation among natural isolates may not linearly dictate the final pathogenic outcome, which is shaped by a complex integrative network. Finally, it is noteworthy that antimicrobial resistance can potentially impact the expression, identification, and detection of virulence factors. The possibility of silent mutations, where genetic markers exist but do not manifest in altered biological phenotypes, cannot be excluded (11).

In summary of the TCS findings, this study reveals a strong association between the expression of *phoQ*, *rstA*, and *ompR* and the hvKP genotype, while finding no such association with carbapenem resistance. These observations come with important limitations. First, the correlative nature of our gene expression data means that causality cannot be established. The elevated expression of TCS may be a consequence, rather than a driver, of the hypervirulent state, or may coexist due to a shared upstream regulator. Second, the sample size for this analysis may limit the detection of subtler associations. Future functional studies are needed to establish mechanistic roles.

### Conclusion

CRKP exhibited lower carriage rates of key virulence genes such as *rmpA*, *iroB*, and *peg-344* but higher rates of *rmpA2* and *ybtS* compared to CSKP, highlighting regional variations in virulence gene evolution. Although hvKP and hmKP prevalence were lower in CRKP, the frequent detection of key virulence genes in CRKP underscores the need for ongoing surveillance due to its dissemination risk. The persistence of CRKP carrying these genes indicates a potential public health threat. In CSKP, virulence genes were strongly correlated, but this correlation was largely absent in CRKP, suggesting that carbapenem resistance may lead to the loss of virulence genes during plasmid transfer. The *Galleria mellonella* assay provided supporting evidence for the enhanced pathogenicity of hvKP and CR-hvKP. Furthermore, TCS genes *phoQ*, *rstA*, and *ompR* were upregulated in hvKP and CR-hvKP, indicating their potential role in virulence regulation. This study has several limitations that should be acknowledged. First, the hypervirulent phenotype was defined molecularly rather than validated by the gold-standard mouse infection model. Second, while strain selection for phenotypic assays considered clinical specimen distribution, it did not systematically encompass the full genetic diversity, although the predominant regional CRKP clone (ST11) was well represented. Most importantly, while our study identifies a strong association between TCS upregulation and the hvKP phenotype in clinical isolates, it remains correlative. The established roles of these TCS from knockout studies in model strains underscore their importance but also highlight the need to validate these mechanisms directly in clinical strain backgrounds to confirm causality and understand their operation in natural settings. Despite these limitations, our findings provide important epidemiological insights and prioritize specific TCS genes for future mechanistic investigation into the interplay between resistance and hypervirulence in *K. pneumoniae*.

## SUPPLEMENTAL MATERIAL

Tables S1 to S3 and Figure S1 spectrum.02030-25-s0001.docx Primers sequence and expression of TCS genes in GSE289556.

Tables S4 and S5 spectrum.02030-25-s0002.xlsx Table S4: Expression counts of TCS genes. Table S5: All reads counts pathogen in GSE289556.

ASM does not own the copyrights to Supplemental Material that may be linked to, or accessed through, an article. The authors have granted ASM a non-exclusive, world-wide license to publish the Supplemental Material files. Please contact the corresponding author directly for reuse.
