# Supplementary material for: Comparative analysis of virulence factors and two-component systems expression in carbapenem-resistant and carbapenem-susceptible Klebsiella pneumoniae isolates at a tertiary hospital in Wenzhou, China
Source: Microbiol Spectr. 2026 May 26;14(7):e02030-25. doi: 10.1128/spectrum.02030-25 (PMC13340050; doi:10.1128/spectrum.02030-25)
Supplement: Tables S1 to S3 and Figure S1 — Primers sequence and expression of TCS genes in GSE289556. [file spectrum.02030-25-s0001.docx]

**Supplementary Material 1**

**Supplementary Table 1** Molecular detection of serotype and virulence gene^[1-3]^

| Gene | Seguence（5′- 3′） | Fragment（bp） | T（℃） |
| --- | --- | --- | --- |
| K1 | F:GGTGCTCTTTACATCATTGC | 1283 | 53 |
|  | R:GCAATGGCCATTTGCGTTAG |  |  |
| K2 | F:CAACCATGGTGGTCGATTAG | 532 | 53 |
|  | R:TGGTAGCCATATCCCTTTGG |  |  |
| K5 | F:TGGTAGTGATGCTCGCGA | 278 | 53 |
|  | R:CCTGAACCCACCCCAATC |  |  |
| K20 | F:CGGTGCTACAGTGCATCATT | 741 | 53 |
|  | R:GTTATACGATGCTCAGTCGC |  |  |
| K54 | F:CATTAGCTCAGTGGTTGGCT | 881 | 52 |
|  | GCTTGACAAACACCATAGCAG |  |  |
| K57 | F:CTCAGGGCTAGAAGTGTCAT | 1037 | 52 |
|  | R:CACTAACCCAGAAAGTCGAG |  |  |
| *rmpA* | F:ACTGGGCTACCTCTGCTTCA | 516 | 53 |
|  | R:CTTGCATGAGCCATCTTTCA |  |  |
| *rmpA2* | F:TGTGCAATAAGGATGTTTACATTAGT | 610 | 53 |
|  | R:TTTGATGTGCACCATTTTTCA |  |  |
| *iucA* | F:GCTTATTTCTCCCCAACCC | 583 | 55 |
|  | R:TCAGCCCTTTAGCGACAAG |  |  |
| *iroB* | F:ATCTCATCATCTACCCTCCGCTC | 235 | 52 |
|  | R:GGTTCGCCGTCGTTTTCAA |  |  |
| *ybtS* | F:GACGGAAACAGCACGGTAAA | 242 | 51 |
|  | R:GAGCATAATAAGGCGAAAGA |  |  |
| *kfu* | F:GGCCTTTGTCCAGAGCTACG | 638 | 58 |
|  | R:GGGTCTGGCGCAGAGTATGC |  |  |
| *peg-344* | F:AAAGGACAGAAAGCCAGTG | 411 | 53 |
|  | R:CAATGACGAGGGGGATAATC |  |  |
| *iut* | F:GGGAAAGGCTTCTCTGCCAT | 920 | 53 |
|  | R:TTATTCGCCACCACGCTCTT |  |  |
| *alls* | F:CATTACGCACCTTTGTCAGC | 764 | 57 |
|  | R:GAATGTGTCGGCGATCAGCTT |  |  |

**Supplementary Table 2**  Quantitative q-PCR primers used in this study.

| Transcription gene | Seguence（5′- 3′） | Reference  or source |
| --- | --- | --- |
| *q-phoQ* | F:ACCCTCACCGATCTCACTC | This study |
|  | R:GGTGTAATTCCCGGCTCAA |  |
| *q-rstA* | F:GCTCACGCCACATAAAACC | This study |
|  | R:ACTCCCACAGCAGATCGAA |  |
| *q-rcsB* | F:CGTCGTTGGCGAGTTTGAAG | This study |
|  | R:CTGAGGATCGCCGGATTGTT |  |
| *q-ompR* | F:CGTATGCTGATGACCGACAA | This study, |
|  | R:TCTTCCGCCGCTTCATTG |  |
| *rpsL* | F:CCGTGGCGGTCGTGTTAAAGA | [4] |
|  | R:GCCGTACTTGGAGCGAGCCTG |  |

**Supplementary Table 3** Capsular serotype distribution between CRKP and CSKP

| Capsular Type | hvKP | CRKP | CSKP |
| --- | --- | --- | --- |
| K1 | hvKP | 4 | 6 |
|  | non-hvKP | 6 | 3 |
|  | total | 10 | 9 |
| K2 | hvKP | 0 | 4 |
|  | non-hvKP | 2 | 0 |
|  | total | 2 | 4 |
| K5 | hvKP | 0 | 0 |
|  | non-hvKP | 1 | 1 |
|  | total | 1 | 1 |
| K20 | hvKP | 1 | 1 |
|  | non-hvKP | 3 | 0 |
|  | total | 4 | 1 |
| K54 | hvKP | 0 | 1 |
|  | non-hvKP | 0 | 2 |
|  | total | 0 | 3 |
| K-non | hvKP | 3 | 9 |
|  | non-hvKP | 130 | 60 |
|  | total | 133 | 69 |


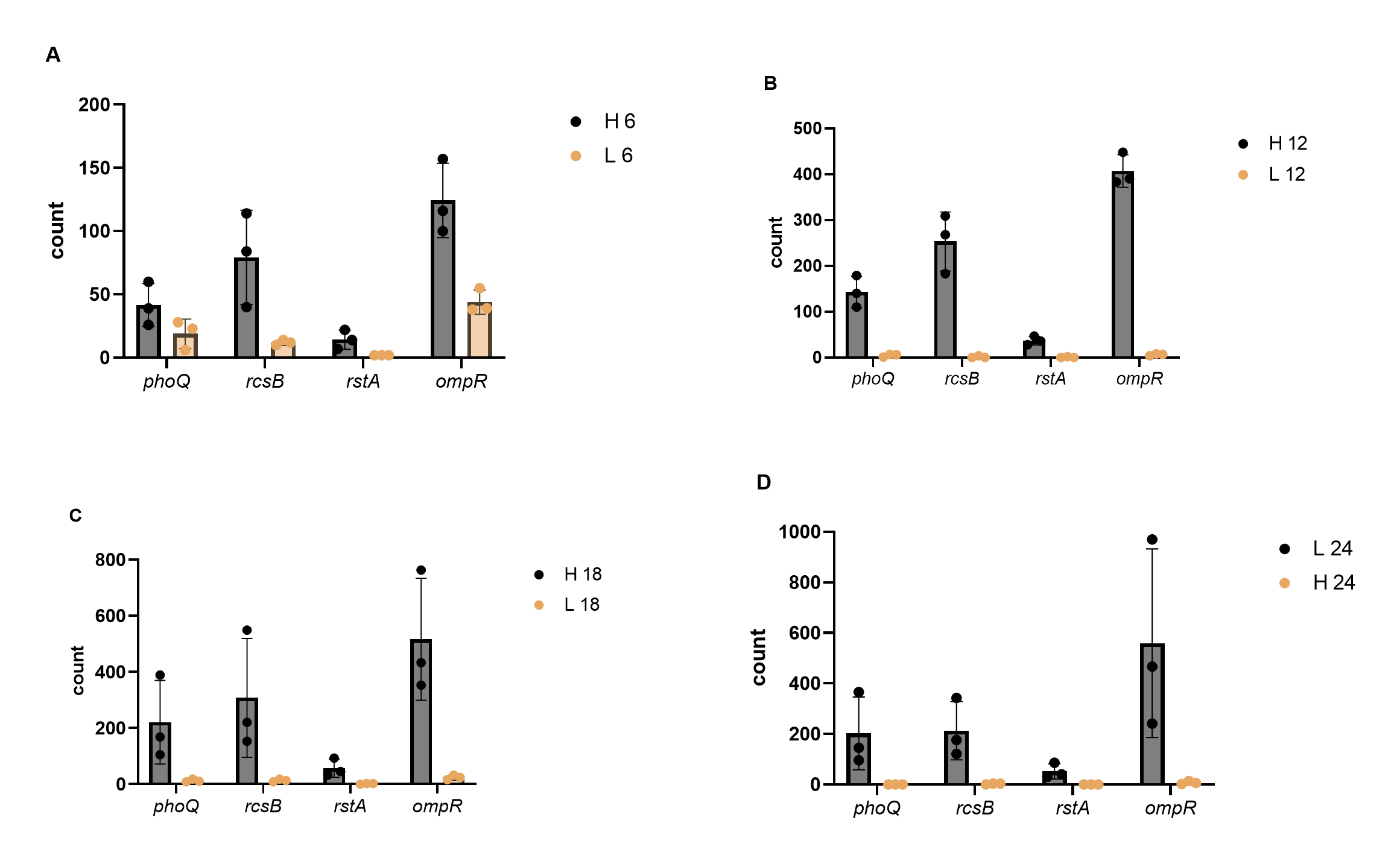


**Supplementary Figure 1. Expression of TCS genes in GSE289556 under high- and low-dose infection conditions.** Normalized read counts for *phoQ*, *rcsB*, *rstA*, and *ompR* were measured at 6h (A), 12h (B), 18h (C), and 24h (D) post-infection under high-dose (3 × 10^8^ CFU) and low-dose ( 7 × 10^6^ CFU) conditions. Bars represent mean values from three biological replicates; error bars indicate standard deviation. All four genes exhibited consistently higher expression in the high-dose group compared to the low-dose group across all time points.

# References

[1]Russo TA, Olson R, Fang CT, Stoesser N, Miller M, MacDonald U, et al. Identification of Biomarkers for Differentiation of Hypervirulent Klebsiella pneumoniae from Classical K. pneumoniae. J Clin Microbiol. 2018;56(9).

[2]Yan Q, Zhou M, Zou M, Liu WE. Hypervirulent Klebsiella pneumoniae induced ventilator-associated pneumonia in mechanically ventilated patients in China. Eur J Clin Microbiol Infect Dis. 2016;35(3):387-96. https://doi.org/10.1007/s10096-015-2551-2.

[3] Zhan L, Wang S, Guo Y, Jin Y, Duan J, Hao Z, et al. Outbreak by Hypermucoviscous Klebsiella pneumoniae ST11 Isolates with Carbapenem Resistance in a Tertiary Hospital in China. Front Cell Infect Microbiol. 2017;7:182.

[4]Jayol A, Poirel L, Brink A, Villegas MV, Yilmaz M, Nordmann P. Resistance to colistin associated with a single amino acid change in protein PmrB among Klebsiella pneumoniae isolates of worldwide origin. Antimicrob Agents Chemother. 2014;58(8):4762-6.
